# Sociodemographic Factors and Comorbidities Including Hyperparathyroidism Are Associated With an Increased Risk of Band Keratopathy: A Population-Based Study in Taiwan

**DOI:** 10.3389/fendo.2022.927513

**Published:** 2022-06-15

**Authors:** Ren-Long Jan, Jhi-Joung Wang, Sung-Huei Tseng, Yuh-Shin Chang

**Affiliations:** ^1^ Graduate Institute of Medical Sciences, College of Health Sciences, Chang Jung Christian University, Tainan, Taiwan; ^2^ Department of Pediatrics, Chi Mei Medical Center, Liouying, Tainan, Taiwan; ^3^ Department of Medical Research, Chi Mei Medical Center, Tainan, Taiwan; ^4^ Department of Anesthesiology, Chi Mei Medical Center, Tainan, Taiwan; ^5^ AI Biomed Center, Southern Taiwan University of Science and Technology, Tainan, Taiwan; ^6^ Department of Ophthalmology, National Cheng Kung University Hospital, College of Medicine, National Cheng Kung University, Tainan, Taiwan; ^7^ Department of Ophthalmology, Chi Mei Medical Center, Tainan, Taiwan

**Keywords:** Band keratopathy, hyperparathyroidism, case-controlled study, sociodemographic factors, Taiwan Longitudinal Health Insurance Database

## Abstract

**Purpose:**

To investigate the association of comorbidities including hyperparathyroidism and sociodemographic factors with band keratopathy.

**Methods:**

This retrospective, population-based, matched case-control study recruited 2,545 patients suffering from band keratopathy. They were selected from the Taiwan National Health Insurance Research Database, based on the International Classification of Diseases, Ninth Revision, Clinical Modification (ICD-9-CM) code 371.43. The control group included 15,270 sex-, age-, and index date-matched non-band keratopathy patients collected from the Taiwan Longitudinal Health Insurance Database 2000. To compare band keratopathy patients with controls, McNemar’s test was used for nominal data and paired t- tests were used for continuous variables. Univariate conditional logistic regression analysis and multivariable conditional logistic regression were used to obtain the odds ratio (OR) and adjusted OR of developing band keratopathy.

**Results:**

Patients with hyperparathyroidism were more likely to develop band keratopathy than controls (OR, 43.5; 95% confidence interval [CI], 23.789–79.544; P < 0.001) even after conditional logistic regression (adjusted OR, 11.28; 95% CI, 5.461–23.33; P < 0.001). Other conditions that increased the odds of scleritis development included systemic diseases such as chronic kidney disease (CKD) and diabetes mellitus (DM) and ocular conditions such as iridocyclitis, phthisis bulbi, and ever silicone oil retention. Regarding sociodemographic factors, >40% of patients with band keratopathy were aged ≥65 years old. Moreover, patients living in Eastern Taiwan and fishermen had higher odds of developing band keratopathy.

**Conclusions:**

Band keratopathy is significantly associated with hyperparathyroidism, CKD, DM, iridocyclitis, phthisis bulbi, and ever silicone oil retention.

## Introduction

Band keratopathy is a chronic degenerative condition which was first described by Dixon in 1848. It is characterized by the formation of whitish to grayish opacities on the corneal surface, especially in the interpalpebral zone (1, 2). The corneal opacities are caused by the precipitation of calcium hydroxyapatite crystals into the superficial layers of the cornea (i.e., the Bowman’s membrane, the epithelial basement membrane, and the basal epithelium) (1, 2). Patients with band keratopathy remain asymptomatic in the early stages of the condition. However, if the opacities extend to the visual axis, the band keratopathy may lead to decreased visual acuity and significant glare. Furthermore, the disruption of the ocular surface due to the accumulation and deposition of the crystals may result in redness, irritation, photophobia, recurrent painful corneal erosion-like symptoms, and vulnerability to corneal ulcers (1).

Histological analysis of affected corneas shows a fine granular calcification in the superficial cornea including the Bowman’s membrane and deep-situated calcium plaques involving the anterior stroma (1, 3). A variety of factors are associated with band keratopathy, including chronic ocular conditions such as uveitis (4, 5), silicon oil retention (6, 7) and phthisis bulbi; and systemic condition such as diabetes mellitus (DM), chronic kidney disease (CKD) (8), elevated serum phosphate levels and increased serum calcium levels that are possibly related to secondary hyperparathyroidism (9–11).

Previous studies reported that hypercalcemia-related ocular manifestations are related to deposition of calcium hydroxyapatite crystals on the ocular surfaces (10, 11). Calcium deposition in the cornea of patients with hyperparathyroidism was first described by Walsh and Howard in 1947 (12). Several previous studies have linked corneal calcium deposition to hyperparathyroidism (11, 13). Some patients were diagnosed as hyperparathyroidism after the finding of calcium deposits in the cornea (14, 15). Several studies also reported that ocular calcium deposition in patients with primary hyperparathyroidism, secondary, or tertiary hyperparathyroidism were related to chronic renal failure (16–19). The mechanism of calcium deposition in the cornea is not understood well, but it may be associated with the precipitates left as tears evaporate, changes in the pH, degeneration and necrosis due to inflammatory diseases, and breakdown of phosphates (1, 20, 21). Primary and secondary hyperparathyroidism may cause high levels of serum calcium and lead to supersaturated concentrations and sediment formation on cornea (9, 11).

The purpose of this study was to investigate the association of hyperparathyroidism, sociodemographic factors, and various comorbidities with band keratopathy and to elucidate the epidemiological features of band keratopathy.

## Materials and Methods

### Patient Data

Data for this study were obtained from the National Health Insurance Research Database (NHIRD) provided by the Taiwan National Health Research Institute. The NHIRD has encrypted identification numbers of patients with demographic data such as date of birth, residential area, and admission and discharge dates. The database also incorporates “International Classification of Diseases, Ninth Revision, Clinical Modification” (ICD-9-CM) codes, and records diagnoses, prescriptions, and procedures; and expenses covered by the National Health Insurance (NHI). This study was exempted from review by the Institutional Review Board of the Chi Mei Medical Center because no identification information were found.

### Selection Criteria

**Figure 1:**
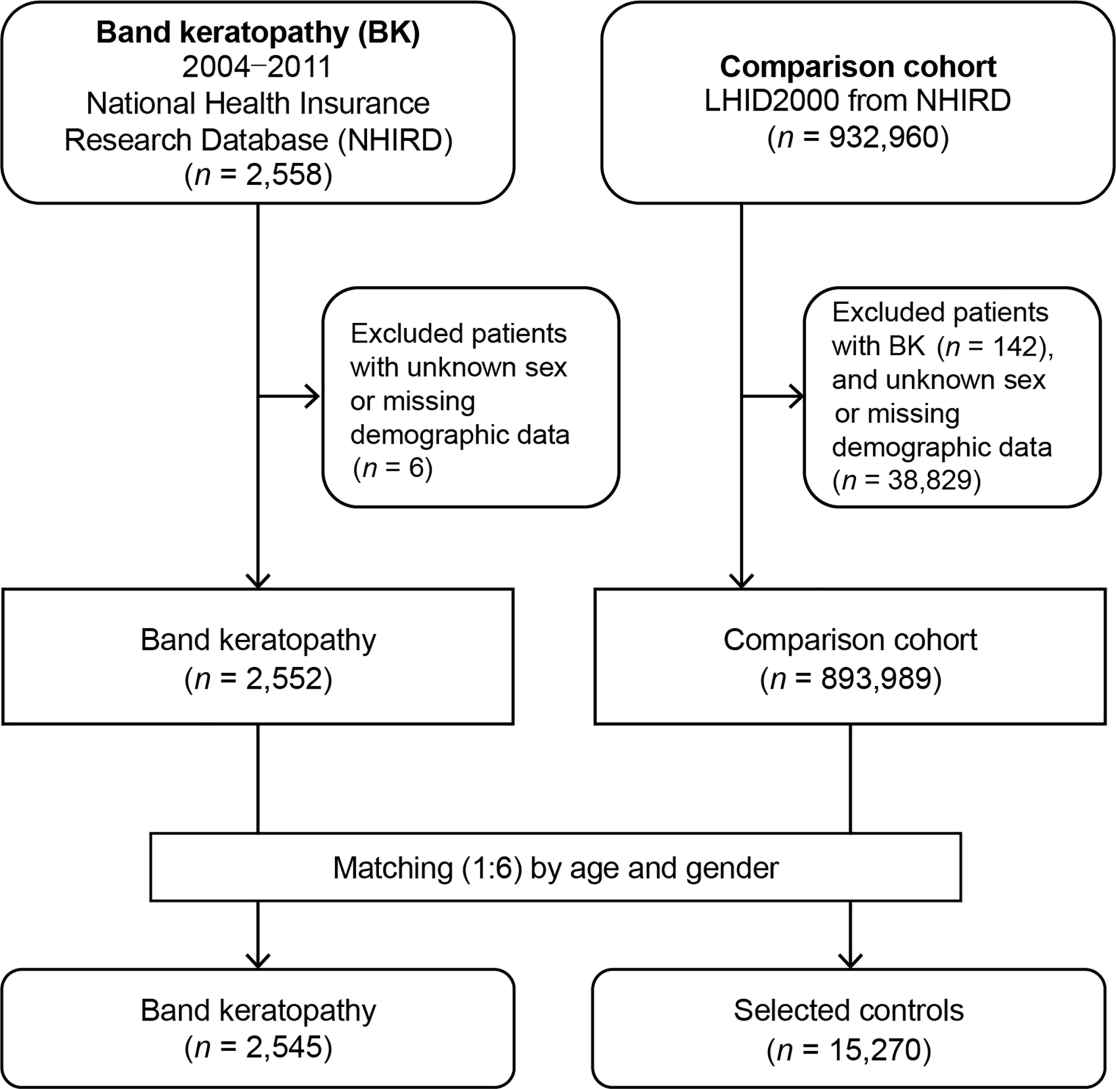
Flowchart demonstrating the enrollment of patients with band keratopathy and controls.

Band keratopathy patients and matched non-band keratopathy control patients were enrolled in this study. The information of patients from both groups was gathered from January 1, 2004, to December 31, 2011. **Figure 1** shows the flowchart of this study. At the beginning, 2,558 patients diagnosed with band keratopathy (ICD-9-CM code 371.43) were enrolled. After excluding patients with missing demographic data and unknown sex, 2,552 patients were left. At the end, 2,545 patients (from the NHIRD) diagnosed with band keratopathy, for whom we found matched controls, were included in the analysis.

For each patient with band keratopathy, six non-band keratopathy controls were randomly selected from the Longitudinal Health Insurance Database (LHID) 2000, which is a subdivision of the NHIRD and comprises the entire data of one million beneficiaries selected in the year 2000. Initially, we recruited 932,818 patients who had not been diagnosed with band keratopathy before the index date. Next, 38,829 patients with unknown sex or missing demographic data were excluded. Controls (n = 15,270) were sex-, age (± 30 days)-, and index date-matched with patients diagnosed without band keratopathy. The index date was defined as the first day the diagnosis of band keratopathy was established. Each patient in both groups was traced and his/her demographic data were recorded from the index date till the date of death or the end of 2011 (for those were still alive after 2011). To determine the medical comorbidities associated with band keratopathy, data regarding comorbidities such as hyperparathyroidism (ICD-9-CM code 252.0: hyperparathyroidism including hyperparathyroidism unspecified [ICD-9-CM code 252.00], primary hyperparathyroidism [ICD-9-CM code 252.01], secondary hyperparathyroidism of non-renal origin[ICD-9-CM code 252.02], and other hyperparathyroidism [ICD-9-CM code 252.08]; ICD-9-CM code 259.3: ectopic hyperparathyroidism; and ICD-9-CM code 588.81: secondary hyperparathyroidism of renal origin); CKD (ICD-9-CM code 585); DM (ICD-9-CM code 250); sarcoidosis (ICD-9-CM code 135); iridocyclitis (ICD-9-CM code 364.0: acute and subacute iridocyclitis, ICD-9-CM code 364.1: chronic iridocyclitis, ICD-9-CM code 364.2 certain types of iridocyclitis, and ICD-9-CM code 364.3 unspecified iridocyclitis); and phthisis bulbi (ICD-9-CM code 360.40: degenerated globe or eye, unspecified and ICD-9-CM code 360.41: blind hypotensive eye) were collected. We used the order code 86214C, which codes for removal of silicon oil, to identify eyes with ever silicone oil retention. Comorbidities were identified based on an ICD-9-CM code being recorded at least once during ambulatory care claims or admittance as an inpatient.

### Statistical Analysis

All statistical analyses were performed using the software SAS 9.4 for Windows (SAS Institute, Inc., Cary, North Carolina, USA). For demographic data, McNemar’s test was used for nominal data analysis and paired *t*-tests were used to analyze continuous variables. Odds ratios (ORs) were obtained using univariate logistic regression analyses. Multivariable logistic regression models (conditional on sex, age, and index date) were created to get the adjusted ORs of developing band keratopathy for patients with various comorbidities. Independent variables included socio-demographic factors (residential city status, geographic residence, income, and employment) and the previously mentioned medical condition of concern. *P* < 0.05 was considered statistically significant.

## Results

### Demographic Data

At the end, 2,545 patients with band keratopathy and 15,270 sex- and age-matched controls who had got medical healthcare services provided by the NHI between 2004 and 2011 were investigated. The mean ages for the band keratopathy patients and controls were 56.77 (standard deviation [SD] 20.98) and 53.79 (18.57) years, respectively (**Table 1**). Of the 2,545 patients with band keratopathy, 255 (10.02%) were aged <25 years, 162 (6.37%) were aged between 25 and 34 years, 231 (9.08%) were aged between 35 and 44 years, 389 (15.28%) were aged between 45 and 54 years, and 1,508 (59.25%) were aged ≥55 years. Among 2,545 patients with band keratopathy, 1,277 (50.18%) were men and 1,268 (49.82%) were women. The incomes of patients with band keratopathy were significantly different from those of controls. The most common approximate income of patients with band keratopathy was < 30,000 New Taiwan dollars (NT$) (2,225; 87.43%), followed by between NT$ 30,000 and NT$ 60,000 (246; 9.67%), between NT$ 60,000 and NT$ 90,000 (53; 2.08%), and > NT$ 90,000 (21; 0.83%) (*P* < 0.001). With regarding to geographic distribution, the most common region of residence of patients with band keratopathy was Northern Taiwan (1,251; 49.16%), followed by the Southern (843; 33.12%), Central (348; 13.67%), and Eastern regions (103; 4.05%) which was significantly different from that of controls (*P* < 0.001). Most patients with band keratopathy resided in rural areas (1,191; 46.80%), followed by a metropolis city (955; 37.52%), and a satellite city (399; 15.68%), which was not significantly different from that in the control group (*P* = 0.202). With regarding to occupation, a significant difference in the distribution was found between the two groups with >50% of patients with band keratopathy having no remarkable occupation; the remaining patients were public servants including military, civil, or teaching staff (440; 17.29%), farmers (390; 15.32%) and fishermen (39; 1.53%) (*P* < 0.01).

**Table 1:** Baseline sociodemographic factors and comorbidities in patients with band keratopathy and controls after matching by age and sex.

	Band keratopathy	Control	*P* value
	N = 2545	N = 15,270	
**Sociodemographic factors**	*n* (%)	*n* (%)	
**Age (years; Mean ± SD)**	56.77 ± 20.98	53.79 ± 18.57	<0.001^a^
**Age (years)**			0.999^b^
< 25	255 (10.02%)	1530 (10.02%)	
25–34	162 (6.37%)	972 (6.37%)	
35–44	231 (9.08%)	1386 (9.08%)	
45–54	389 (15.28%)	2334 (15.28%)	
55–64	461 (18.11%)	2766 (18.11%)	
≥ 65	1047 (41.14%)	6282 (41.14%)	
**Sex**			0.999^b^
Male	1277 (50.18%)	7662 (50.18%)	
Female	1268 (49.82%)	7608 (49.82%)	
**Income**			<0.001^b^
< NT$ 30,000	2225 (87.43%)	12868 (84.27%)	
NT$ 30,000–60,000	246 (9.67%)	1860 (12.18%)	
NT$ 60,000–90,000	53 (2.08%)	370 (2.42%)	
> NT$ 90,000	21 (0.83%)	172 (1.13%)	
**Geographical region of Taiwan**			<0.001^b^
Northern	1251 (49.16%)	7121 (46.63%)	
Central	348 (13.67%)	1958 (12.82%)	
Southern	843 (33.12%)	5818 (38.1%)	
Eastern	103 (4.05%)	373 (2.44%)	
**Residential city status**			0.202^b^
Metropolis	955 (37.52%)	5555 (36.38%)	
Satellite	399 (15.68%)	2281 (14.94%)	
Rural	1191 (46.8%)	7434 (48.68%)	
**Occupation**			<0.001^b^
Public servant	440 (17.29%)	3183 (20.84%)	
Farmer	390 (15.32%)	2305 (15.09%)	
Fisherman	39 (1.53%)	189 (1.24%)	
Other	1676 (65.85%)	9593 (62.82%)	
**Comorbidities**			
Hyperparathyroidism	87 (3.42%)	12 (0.08%)	<0.001^b^
Chronic kidney disease	578 (22.71%)	439 (2.87%)	<0.001^b^
Diabetes mellitus	887 (34.85%)	2278 (14.92%)	<0.001^b^
Sarcoidosis	2 (0.08%)	2 (0.01%)	0.073 ^b^
Iridocyclitis	171 (6.72%)	116 (0.76%)	<0.001^b^
Phthisis bulbi	257 (10.1%)	13 (0.09%)	<0.001^b^
Ever silicon oil retention	26 (1.02%)	1 (0.01%)	<0.001^b^

^a^Paired t-test; ^b^McNemar’s test.

NT$, New Taiwan dollars; SD, standard deviation.

Patients with band keratopathy exhibited a significantly higher prevalence of systemic diseases such as hyperparathyroidism (87; 3.42%), CKD (578; 22.71%), and DM (887; 34.85%) than controls (*P* < 0.001; **Table 1**). There was evidence of significant differences regarding presence of ocular conditions such as iridocyclitis (171; 6.72%), phthisis bulbi (257; 10.1%), and ever silicone oil retention (26; 1.02%) between patients with band keratopathy and controls (*P* < 0.001; **Table 1**). There was no significant difference with regard to incidence of sarcoidosis among patients with band keratopathy (2; 0.08%) and controls (*P* = 0.073).

### Associated Risk Factors

Socio-demographic factors (including wages, geographic residence, residential city status, and employment) of band keratopathy patients and matched controls were analyzed using univariate logistic regression analyses and a multiple logistic regression model. The odds of developing band keratopathy for patients with income between NT$ 30,000 and NT$ 60,000, NT$ 60,000 and NT$ 90,000, and > NT$ 90,000 were not significantly increased when compared with those with income < NT$ 30,000 after adjusting for other confounders. Regarding the patients’ geographic residences, odds of band keratopathy was significantly higher for patients residing in Eastern Taiwan (OR, 1.583; 95% confidence interval [CI], 1.261–1.988; P < 0.001) than for those in Northern Taiwan, and residence in Eastern Taiwan remained a significant risk factor (for developing band keratopathy) after using conditional logistic regression analysis (adjusted OR, 1.671; 95% CI, 1.277–2.187; P < 0.001). Patients whose employment was fishing had a significantly higher prevalence of band keratopathy (OR, 1.546; 95% CI, 1.077–2.221; P = 0.018) compared to public servants (teaching, military, or civil staffs), and the fishing profession remained a significant risk factor after conditional multivariable logistic regression analysis (adjusted OR, 1.580; 95% CI, 1.030–2.423; *P *= 0.036) (**Table 2**).

**Table 2:** Odds ratios and adjusted odds ratios for various sociodemographic factors and comorbidities associated with band keratopathy.

	Odds Ratio^a^	*P* value	Adjusted Odds Ratio^b^	*P* value
	(95% CI)		(95% CI)	
**Sociodemographic factors**				
**Income**			
< NT$ 30,000	1.00		1.00	
NT$ 30,000–60,000	0.732 (0.631–0.849)	<0.001	0.894 (0.735–1.089)	0.266
NT$ 60,000–90,000	0.783 (0.58–1.056)	0.109	1.146 (0.793–1.656)	0.469
> NT$ 90,000	0.669 (0.421–1.062)	0.088	1.225 (0.732–2.049)	0.440
**Geographical region of Taiwan**				
Northern	1.00		1.00	
Central	1.012 (0.889–1.152)	0.858	1.013 (0.864–1.188)	0.874
Southern	0.824 (0.75–0.905)	<0.001	0.84 (0.751–0.939)	0.002
Eastern	1.583 (1.261–1.988)	<0.001	1.671 (1.277–2.187)	<0.001
**Residential city status**				
Metropolis	1.00			
Satellite	1.018 (0.896–1.156)	0.785		
Rural	0.932 (0.85–1.022)	0.132		
**Occupation**				
Public servant	1.00		1.00	
Farmer	1.325 (1.122–1.564)	<0.001	1.198 (0.957–1.501)	0.115
Fisherman	1.546 (1.077–2.221)	0.018	1.580 (1.030–2.423)	0.036
Other	1.338 (1.181–1.515)	<0.001	1.136 (0.955–1.352)	0.151
**Comorbidities**				
Hyperparathyroidism	43.5 (23.789–79.544)	<0.001	11.287 (5.461–23.33)	<0.001
Chronic kidney disease	10.55 (9.152–12.16)	<0.001	7.357 (6.258–8.649)	<0.001
Diabetes mellitus	3.512 (3.173–3.887)	<0.001	2.617 (2.324–2.947)	<0.001
Sarcoidosis	6 (0.845–42.594)	0.073		<0.001
Iridocyclitis	9.559 (7.486–12.206)	<0.001	9.509 (7.183–12.589)	<0.001
Phthisis bulbi	139.556 (76.323–255.179)	<0.001	141.968 (75.296–267.676)	<0.001
Ever silicon oil retention	156 (21.169–1149.582)	<0.001	118.612 (14.595–963.938)	<0.001

^a^Odds ratio was obtained from a univariate conditional logistic regression analysis; ^b^Adjusted odds ratio was calculated from a multivariable conditional logistic regression model that was conditioned on age-group, sex, and the year of index date.

NT$, New Taiwan dollars; CI, confidence interval.

The associations of several possible comorbidities with band keratopathy were also analyzed using univariate and multiple logistic regression analyses (**Table 2**). Patients with hyperparathyroidism had significantly higher ORs of being diagnosed with band keratopathy (OR, 43.5; 95% CI, 23.789–79.544; P < 0.001), and the OR remained high even after conditional logistic regression analysis (adjusted OR, 11.287; 95% CI, 5.461–23.33, P < 0.001). Patients with CKD and DM had higher ORs of being diagnosed with band keratopathy before (OR, 10.55; 95% CI, 9.152–12.16; P < 0.001 and OR, 3.512; 95% CI, 3.173–3.887; P < 0.001, respectively) and after adjusting for other confounders (adjusted OR, 7.357; 95% CI, 6.258–8.649; P < 0.001; adjusted OR, 2.617; 95% CI, 2.324–2.947; P < 0.001, respectively) (**Table 2**). Patients with ocular conditions such as iridocyclitis, phthisis bulbi, and silicone oil retention had a significantly higher ORs of being diagnosed with band keratopathy (OR, 9.559; 95% CI, 7.486–12.206; P < 0.001; OR, 139.556; 95% CI, 76.323–255.179; P < 0.001; and OR, 156; 95% CI, 21.169–1149.582; P < 0.001, respectively), and the ORs remained high after conditional multivariable logistic regression analysis (adjusted OR, 9.509; 95% CI, 7.183–12.589; P < 0.001; adjusted OR, 141.968; 95% CI, 75.296–267.676; P < 0.001; and adjusted OR , 118.612; 95% CI , 14.595–963.938; P < 0.001, respectively) (**Table 2**).

## Discussion

To the best of our knowledge, our study is the largest population-based, case-control study that explores the association between socio-demographic factors and common comorbidities (like hyperparathyroidism) with band keratopathy. Our analyses identified several key findings. First, > 40% of the patients with band keratopathy in Taiwan were aged ≥ 65 years and the male to female distribution was equal. Second, the odds of developing band keratopathy varied according to several sociodemographic factors. Patients living in Eastern Taiwan and fishermen had higher odds of developing band keratopathy. Third, the presence of some comorbidities significantly influenced the odds of developing band keratopathy, and patients with hyperparathyroidism had significantly higher odds of developing band keratopathy (adjusted OR, 11.287; 95% CI, 5.461–23.33; P < 0.001, **Table 2**).

Of the 2,545 patients with band keratopathy included in this study, 1508 (59.25%) were aged ≥ 55 years. Possible explanations for this finding include the observation that corneal calcium deposition may be a gradual process and correlated with increasing age. Moreover, many systemic diseases such as CKD and DM, which have a high prevalence in older individuals, were risk factors for band keratopathy. In this study, the male to female ratio of patients with band keratopathy was 1.01:1. Thus, no sex preponderance was demonstrated.

Regarding socio-demographic factors, we found statistically significant associations between band keratopathy and patients living in Eastern Taiwan compared with those living in Northern Taiwan (adjusted OR, 1.671; 95% CI, 1.277–2.187; P < 0.001, **Table 2**), and patients whose occupation was fishing compared with those in public service (adjusted OR, 1.580; 95% CI, 1.030–2.423; P = 0.036, **Table 2**). It is worth noting that Eastern Taiwan has a long coastline, and most people living in Eastern Taiwan make a living as fishermen. The higher rate of band keratopathy diagnosis in Eastern Taiwan and fishermen in our study, may be attributed to the humid and windy coastal climate with full sunlight in Eastern Taiwan, which may enhance calcium precipitation compared with that in other regions of Taiwan. Individuals with a lower income did not have significantly higher odds of developing band keratopathy, which implied that patients with band keratopathy did not have limitation in employment due to limitation of visual acuity by the disease.

In this study, patients with hyperparathyroidism including primary hyperparathyroidism and secondary hyperparathyroidism due to non-renal or renal impairments had a remarkably higher OR for band keratopathy development (adjusted OR, 11.287; 95% CI, 5.461–23.330; P < 0.001). Previous studies have reported corneal calcium deposition in patients with secondary hyperparathyroidism with hypercalcemia secondary to chronic renal failure (10, 16) or with normal serum calcium but with vitamin D insufficiency (22). These deposits were identified as calcium hydroxyapatite crystals (11), which is primarily composed of calcium and phosphate (23). Possible factors that trigger the formation of hydroxyapatite crystals include abnormalities in calcium, vitamin D, and parathormone metabolism (15, 19); a change in pH value over the interpalpebral ocular surface; and an increase in the local concentrations of calcium and phosphate ions (2).

CKD is an independent risk factor for band keratopathy after adjusting for other confounders (adjusted OR, 7.357; 95% CI, 6.258–8.649; P < 0.001, **Table 2**). This finding is consistent with that in previous studies (8, 24). In a study of 94,039 patients with CKD, Weng et al. showed that CKD was an independent risk factor of band keratopathy (adjusted hazard ratio, 11.56 [95% CI, 7.70–17.35]) in the total cohort (8). The increased risk for band keratopathy in patients with CKD may be explained by the elevated serum phosphate level (25), increased serum calcium level (24, 26, 27), and greater frequency of long-standing eye-drop instillation due to irritable red eyes and ocular hypertension (28, 29).

In this study, DM was another significant risk factor for band keratopathy (adjusted OR, 2.617; 95% CI, 2.324–2.947; P < 0.001, **Table 2**). No previous study has demonstrated a relationship between DM and band keratopathy. Several studies showed that the prevalence ocular surface problems such as recurrent corneal erosions and corneal ulcers is significantly higher in patients with DM (30, 31). Compromised corneal surface, delayed epithelial wound healing, and corneal epithelial basement membrane abnormalities are common in patients with DM (32–34) and may lead to an increased risk of recurrent corneal erosions and corneal ulcers. These corneal conditions may result in the cornea being vulnerable to sub-epithelial precipitation of calcium hydroxyapatite in patients with DM and may be important reasons for the increased risk of band keratopathy in patients with DM.

Our findings showed that iridocyclitis is indeed an independent risk factor of band keratopathy development (adjusted OR, 9.509; 95% CI, 7.183–12.589; P < 0.001, **Table 2**). This finding is consistent with that in previous reports, which showed that band keratopathy was a common ocular complication (19.2%–38%) in patients with Juvenile idiopathic arthritis-associated uveitis (4, 35, 36). The exact pathophysiology of band keratopathy is unknown, but it may be associated with phosphate breakdown due to pH changes on the ocular surface. Alkaline changes in the palpable ocular surface may occur due to precipitation as tears evaporate or degeneration and necrosis due to chronic ocular inflammatory diseases (1, 2).

Regarding the association between ocular surgery and band keratopathy, the results of our regression analysis show that silicon oil tamponade is an important risk factor for band keratitis (**Table 2**). Silicone oil has been widely used as a vitreous substitute in advancement of vitreoretinal surgical techniques. Silicone oil is either removed 3–6 months postoperatively or long-term silicone tamponade is performed depending on various diseases and situations. The observation is the same as that in several studies, which demonstrated that silicone oil tamponade is an important risk factor for band keratopathy (6, 7, 37) even after removal (38). Although the mechanism of band keratopathy is multifactorial, many studies have shown that silicone oil plays a significant role in the development of band keratopathy (7, 37) and that the tissue toxicity of silicone oil or pH changes due to from decreased flow across the corneal tissue may be associated with band keratopathy development (39).

Our study had several strengths. It is the largest study till date to focus on patients with band keratopathy, with 2,545 cases identified in the NHIRD database. The claims data of the NHIRD are recorded electronically rather than depending on patient self-reporting of medical conditions, which reduces the recall bias. Since our data were based on a nationwide population-based dataset, the selection bias regarding referral centers was obviated. In addition, this case-control study incorporated longitudinal data from 10 years on various sociodemographic factors and comorbidities in patients and controls. It is worth noting that these sociodemographic factors and comorbidities including hyperparathyroidism were recognized as potential confounding factors and appropriately adjusted when assessing the OR in patients with band keratopathy.

This study had several limitations. First, the presence of band keratopathy among those identified as patients or the absence of band keratopathy in controls was based on claims data and was not confirmed by assessment of clinical records. In addition, the diagnosis of band keratopathy and other comorbidities based on ICD-9-CM codes may lead to disease misclassification. We could not assess whether the management of blood sugar level and glycosylated haemoglobin influenced the risk of developing band keratopathy, because the insurance claims data did not include information on the current blood sugar value or haemoglobin A_1_C level. Finally, there were no data to confirm that controls had not been diagnosed with band keratopathy before January 1996 due to the absence of information before 1996, which may result in potentially compromised findings.

In conclusion, this study described some socio-demographic factors (such as living in Eastern Taiwan and fishing jobs) associated with an increased risk of developing band keratopathy. It is important to notice that after adjusting for socio-demographic factors and some comorbidities such as CKD, DM, iridocyclitis, phthisis bulbi, and silicone oil retention, patients with hyperparathyroidism had a significantly higher risk of developing band keratopathy than controls. To the best of our knowledge, our study is the largest study to show an association between hyperparathyroidism and band keratopathy development. This associations should be clarified in future studies, to enhance the understanding of the epidemiology and pathophysiology of band keratopathy.

## Data Availability Statement

The original contributions presented in the study are included in the article/supplementary material. Further inquiries can be directed to the corresponding author.

## Author Contributions

Conceptualization, R-LJ and Y-SC; Formal analysis, R-LJ and Y-SC; Methodology, R-LJ and Y-SC; Resources, J-JW; Software, J-JW; Writing – original draft, R-LJ and Y-SC; Writing – review and editing, S-HT and Y-SC. All authors contributed to the article and approved the submitted version.

## Author Disclaimer

The conclusions and interpretations incorporated here do not represent those of the Bureau of National Health Insurance, Department of Health, or National Health Research Institutes.

## Conflict of Interest

The authors declare that the research was conducted in the absence of any commercial or financial relationships that could be construed as a potential conflict of interest.

## Publisher’s Note

All claims expressed in this article are solely those of the authors and do not necessarily represent those of their affiliated organizations, or those of the publisher, the editors and the reviewers. Any product that may be evaluated in this article, or claim that may be made by its manufacturer, is not guaranteed or endorsed by the publisher.

## Abbreviations

CI, confidence interval; DM, diabetes mellitus; CKD, chronic kidney disease; ICD-9-CM, International Classification of Diseases, Ninth Revision, Clinical Modification; LHID 2000, Longitudinal Health Insurance Database 2000; NHI, National Health Insurance; NHIRD, National Health Insurance Research Database; NHRI, National Health Research Institute; NT$, New Taiwan dollars; OR, odds ratio; SD, standard deviation
